# Anæsthesia

**Published:** 1864-03

**Authors:** 


### Selections.

### ANÆSTHESIA.

(BY A LOVER OF TRUTH.)

*Priority of conception and experimentation. Is* Horace Wells *entitled to the credit of both? Did he verify his discovery and make it practical? Did he ever abandon it? Was he intercepted? And, if so, by whom, and under what circumstances? Was such interception fair, and the means used, honorable and just? Shall trickery and fraud prevent the transmission of his name to posterity with all the éclat which sach a vastly important discovery is adapted to confer.*

Writing as a Lover of Truth and Justice, I am constrained to treat of these topics, and fortunately the facts can be so clearly ascertained and substantiated by such an abundance of unquestionable proof, that no candid upright man can hesitate for a moment as to the conclusions to which he should come.

Here I would observe that Wm. T. G. Morton has caused the priority of conception and experimentation to be adjudicated by a competent tribunal in favor of Horace Wells. At the first session of the Thirty-second Congress (which convened at Washington on the first Monday of December, 1851), Morton proffered a memorial to the House of Representatives asserting his claims, and praying for what he has so long prayed in vain. This memorial was referred to a select committee, at the head of which was placed the gallant and accomplished Col. Wm. H. Bissell, afterward Governor of Illinois, but since deceased. Before this committee Morton and Jackson appeared with their counsel, and with their exhibits and proofs, but, alas! Horace Wells had been some years in his grave, and he had left his wife and a son of tender years penniless, and they could neither employ counsel nor collect or bring forward the necessary proofs. Wells, himself had, in his lifetime, written and published a small pamphlet in vindication of his claims, which is now before me. It is distinguished by all the fairness, candor, evident truthfulness, and good sense which ever characterized his life and conversation. To this is annexed a considerable amount of proof, and is dated at Hartford, March 30th, 1847. It is well adapted to arrest attention, and to make impressions quite favorable to his pretensions, but does not do full justice to the subject. How was this to be expected from Wells, who had neither the education nor the practice indispensable to successful authorship. I infer from Col. Bissell's report, that he had this pamphlet before him, probably transmitted by some friend of the family. This, however, was not sufficient to prevent his investigations having an *ex parte* character so far as the Wells' claim was concerned. This was not the fault of Col. Bissell, though it may have been a serious misfortune to the family of Wells, and to the cause of rectitude and truth.

Hence the worthy Colonel had little to consider, but the conflicting pretensions of Morton and Jackson, and "the conjunction" of their labors and efforts had been carefully concealed from him by mutilating an important document. Had he known this fact, would he have toiled through many pages of argumentation, to show how the balance between them stood? Would he not have exclaimed, No! no! gentlemen! You have *sworn* yourselves into the same side of the scales! There is no question of preponderance the one way or the other between you. Either from you conjointly, or from Wells, anæsthesia must have emanated. Cease, therefore, your bickerings and consider how the pretensions of your co-partnership stand as against the claims of Wells. This would have brought those claims to the particular notice of the honorable member, and perhaps, he might have given them a favorable consideration, or perhaps, he might have awarded to the *twin* investigators of anæsthesia, this great inheritance of fame, and concluded that "beautiful in their lives, in their deaths they should not be divided." This certainly would have been sublime, though it is said that "there is only one step from the sublime to the ridiculous." Poor Wells and all his labors might, in such case have passed off into nonentity. But Col. Bissell was not prepared to admit the justice or propriety of consigning him to such a fate. It is obvious that he thought the pretensions advanced in his favor, were far from being frivolous. This will appear from the following extract from his report:

"The evidence presented with Dr. Wells' claim, shows that *dental operations were in several instances performed without pain*, by Dr. Wells, under the influence of the nitrous oxyd which had before been known in some cases to produce a total or partial asphyxia. It appears also that the vapor of sulphurous ether was thought of, discussed, and finally rejected by him, while the total abandonment of the use of the nitrous oxyd, and indeed of every other agent shows that Wells' experiments were, on the whole, unsuccessful. He engaged in the search, and failed to find the object of his pursuit. He attempted and endeavored *assiduously* to carry out the idea to practical results, but was not successful." * * * "He had the great idea of producing insensibility to pain, but he did not verify it by successful experiments." * * * "*But he had the signal merit of reviving the investigation and probably of hastening the discovery.*"

This would seem to settle some points conclusively in favor of the Wells' claim. Col. Bissell accords to him the credit of 1. Priority of conception. 2. Priority of experimentation. 3. Priority of verification. "*Dental operations*," he says, "*were in several instances performed without pain*, by Dr.

Wells, under the influence of the nitrous oxyd." 4. "He had the great idea of producing insensibility to pain." 5. "He endeavored *assiduously* to carry out the idea." 6. "He had the signal merit of reviving the investigation," (referring, I suppose, to some experiments tried by Sir Humphrey Davy toward the close of the last century,) and 7. "Perhaps of hastening the discovery." Ah! *Signal merit* (perhaps) *of hastening the discovery!*" Here would seem to be a little significant squinting at the source of the information on which Jackson and Morton acted, in causing Frost's tooth to take a painless leap out of his jaw. Recollect "*murder will out*," though often detected by very slight circumstances.

But Col. Bissell felt constrained after all to reject and set aside the Wells' claim, for he says, 1. "The vapor of sulphuric ether was thought of, discussed, and finally rejected." 2. "He abandoned the use of nitrous oxyd," and 3. "Of every other agent," and 4. "His experiments on the whole, were unsuccessful." But it is certain that he was not wholly unsuccessful, for Col. Bissell admits that he realized his object "in several instances" of dental operation, and that he could not have abandoned the subject early, is certain, for the Colonel also adds that "he *assiduously* endeavored to carry out the idea," and that he abandoned it at all or ever thought of doing so, will be news at Hartford, Conn., and news too of a very apocryphal character. I do not know of any more striking illustration than is to be found in this extract of the misconceptions which may be exhibited by a trusty, just man, in writing, *currente calamo* on a subject which he has not fully before him, and which he does not perfectly understand.

I do not hesitate to declare a confident belief that a proper presentation of the subject, will bring every upright mind to conclusions directly the reverse of those written down by Col. Bissell, so far as they are adverse to the Wells' claim. I affirm that he never did abandon the nitrous oxyd while living; that it never did, in his hands, produce asphyxia or anything approaching it; that he prosecuted practical anæsthesia "assiduously" (to use Col. Bissell's appropriate word), and made all the progress toward a general introduction of the new practice which the astonishing and almost miraculous character of his developments would permit, and furthermore that his name would long since have been blazoned to the world as the true author of anæsthesia and a benefactor of his race, had he not been intercepted by arts and practices alike insidious and detestable.

My proofs are full, unexceptionable, and conclusive, so that all must believe except such as "would not be persuaded though one rose from the dead." It is in my power to produce nearly a whole city under oath, and to bring forward the testimony of a Right Rev. Bishop of the Episcopal Church (now like Wells gone to his final account); several doctors of divinity and other members of the clerical profession, most of the physicians and surgeons and all or nearly all of the practitioners of the dental art, one or more of the learned professors of Washington College, Members of the United States Senate and House of Representatives, counsellors at law, merchants, manufacturers, mechanics, artizans, and many of the fairer part of God's creation—ladies of refinement and of the first respectability,—in short, a whole cloud of witnesses, most of whom will speak of their own personal knowledge, and the residue of facts which fell under their personal observation.

But as I have not the slightest interest in this matter, and write only as a "Lover of Truth and Justice," desiring to uphold the right, and taking a deep interest in the welfare of a poor, defenceless and much injured family, it is hardly to be expected that I should incur all the labor and expense of examining witnesses who have already spoken to the facts, and of taking testimony which is now before the public. No doubt I might procure much cumulative evidence, but as we have an abundance now on hand, that would be a work of supererogation.

I shall therefore content myself with bringing to the notice of your readers a thin volume, or (if you please) a pamphlet named "Anæsthesia," published in 1858, by Mr. Truman Smith, late a member of the United States Senate, which contains proofs collected by him in the fall of 1852, with a view to lay the same before that body, if needful. It turned out, however, otherwise, for reasons not necessary to be here explained, and the proofs in manuscript remained in the hands of Mr. Smith till 1858, when he gave them in the form already indicated to the public. In this publication Mr. Smith has digested and arranged the evidence under appropriate heads so as to exhibit separately and distinctly all the essential elements of the Wells' case, and has accompanied the same with remarks and suggestions such as he deemed appropriate and just. I shall not be so absurd as to ask you to republish this vast mass of testimony or any considerable portion of it, but I presume your readers will indulge me in presenting a synopsis of the contents with extracts from the testimony so far as may be necessary to show how conclusively the Wells' case is made out.

1. Mr. Smith opens the subject with considering "what constitutes a discovery in respect to this and other analagous subjects." "It is believed" (he says) "to lay the foundation for just pretensions, it is indispensable that the party should have formed a distinct conception of anæsthesia, and should have at least, substantially attained that end by good and satisfactory means. If to both of these elements he can add also that of priority he must be regarded as the true discoverer, and his position as such, will appear the stronger if he has given early and full publicity to his experiments." Mr. Smith then adds that "it is not necessary to constitute a true discoverer in respect to any matter of nature or art that he should have resorted in the first instance to the best means of development." And he insists that all that is required is that "the agency should be competent to demonstrate the value and importance of the new idea;" and this being done, it is to be expected (as he remarks justly) "that many other minds, active, ingenious, and inventive, will be directed into the same channel of inquiry, pursuing the object proposed by the original discoverer or inventor, with vigor and success, suggesting improvements, contriving substitutes, and introducing new agents which carry the discovery, invention, or art, far beyond the point at which it was kept by the real author of the movement." I do not doubt your readers will accept these views as sound, and they should be borne incessantly in mind by all who desire to arrive at a proper result on this entire subject.

2. Mr. Smith next takes the ground that the nitrous oxyd and the vapor of sulphuric ether had long been known to produce when inhaled, similar effects, to occasion excitement, a species of intoxication, and some degree of insensibility, and it being ascertained that the nerves of sensation could by the inhalation of the former, be so completely paralyzed that dental and surgical operations could be performed without pain, there would be no merit whatever in substituting the vapor of ether for the nitrous oxyd, and that it would be a perversion of terms to call the authors of such substitution (which any tyro of chemistry might suggest) a discoverer, and the rankest injustice to allow him to usurp the place of and reap all the rewards and honors which should be accorded to the party who originated the system. Mr. Smith produces a letter from the learned professor Thomas D. Mutter of your city, from which I make the following extract:

"Now if it can be shown that Mr. Wells (I do not say that he did, as this is a question of which I know nothing positively) first demonstrated usefully and practically, the fact that operations can be performed without pain in consequence of the inhalation by the individual of some gaseous substance like nitrous oxyd gas or the vapor of ether, *then beyond all question* is he entitled to the honor and reward of having established one of the most valuable facts in the science of surgery." * * * * * * * "The subsequent introduction by others of agents of a similar character, even although more efficient than those first employed, does not at all diminish his claim to having established the great fact"

Professor Abner Jackson, of Trinity College, Hartford, Conn., says, "the person who first applied either nitrous oxyd gas, sulphuric ether, or chloroform, should, in my opinion, be regarded as the true discoverer, inasmuch as the use of the others would be a natural sequence."

Professor Willard Parker, of the city of New York: "I further say, it being known that nitrous oxyd would produce anæsthesia in surgical operations, it would suggest to any one having any knowledge of the two substances that sulphuric ether would produce the same effect, and the substitution of the ether for the gas, does not in my opinion merit the name of discovery."

Professor John W. Francis: "The well known sedative effects of sulphuric ether and others might readily suggest to the scientific mind their substitution for the nitrous oxyd gas, and the application of any one of these agents may be fairly recognized as the primary discovery."

Mr. Smith produces several other depositions to the same effect, but it is unnecessary to make further quotations. Let this feature of the case be borne distinctly in mind as it must in connection with facts hereafter to appear, prove fatal to the pretensions of Jackson and Morton whether taken jointly or separately.

3. Mr. Smith then arrives at an era of vast interest to humanity, when the anæsthetic idea was not only conceived and announced by its author, but was developed and brought out by experimentation, plain, unequivocal and complete. I have already given a rapid sketch of what occurred at Prof. Colton's lecture on the evening of the 10th December, 1844, and what at a brother dentist's office the next day. That dentist was Mr. John M. Riggs, of Hartford, and known to me to be a gentleman of respectability. Mr. Smith exhibits vividly the whole of the remarkable transaction which commenced on the evening of the 10th, and which was consummated on the 11th, by producing the testimony of Mr. G. Q. Colton (now of No. 22 Bond street, New York), Mr. John M. Riggs, Mrs. Elizabeth Wells, Mr. Samuel A. Cooley, and Mr. David Clarke, and shows that if these witnesses are to be believed under oath, that there was not only a conception by Wells of the true anæsthetic idea, but verification of it by an unequivocal experiment on himself—that he then and there caused, after inhaling the nitrous oxyd, one of his large molar teeth to be extracted without the slightest pain, and thus detected and brought to light one of the most wonderful secrets of nature—*the first* to *conceive*, the *first to experiment*, and the *first to verify beyond question or doubt! These, these are the facts!* established by so many witnesses! It is useless to produce their depositions or to make extracts. Let those who wish to look more closely into this matter, recur to Mr. Smith's Anæsthesia from p. 18 to p. 25.

But what a wonderful contrast is there between the Wells case and the Morton case as to the extent of the proofs adduced to show their character. In the latter only one witness so far as I know has ever been examined (Dr. Hayden) who held the lamp while Morton extracted Frost's tooth after he (F.) had inhaled and been brought under the influence of the vapor of sulphuric ether, and yet no one pretends to doubt or dispute the fact. How audacious then is it for any man to attempt to controvert the success of the Wells experiment, proved and established as it is by so many witnesses, not one of whom has the slightest interest in the subject, if Mrs. Wells be excepted.

I will here remark, that Mr. G. Q. Colton having had the good fortune to assist at the first genuine anæsthetic operation ever performed on earth, immediately resumed his lectures, and continued the same until within a recent period, all the while exhibiting the nitrous oxyd to illustrate chemical principles, and having nothing to do with anæsthesia as such. Whether facts have not been recently developed by his agency, most material to be considered in this connection, will be a subject of inquiry hereafter.

4. The great discovery having been made, Dr. Wells and his brother dentists immediately introduced into their practice, at Hartford, the nitrous oxyd as an anæsthetic agent, and continued such use during the interval between December 11th, 1844, and September 30th, 1846, and long after. Mr. Smith brings forward many depositions on this point, but extracts from a few must suffice.

Dr. John M. Riggs: "We were so elated by the success of this experiment" (that is to say the operation on Dr. Wells of December 11th,) "that we immediately turned our attention to the extraction of teeth by means of this agent, and continued to devote ourselves to this subject for several weeks almost exclusivly." * * * * * "Dr. Wells continued to use the gas freely in the practice of dentistry during the remainder of that year and the year following, and at all times when he was in the practice of his profession. I, myself, also used it as people demanded it, which they ordinarily did." * * * * "I find on reference to my books, that this agent was used by me in extracting teeth up to November 2d, 1846, which is my last charge. Since that time I have used chloroform generally, when my patients requested anything." * * * "I was in the habit, for greater ease in furnishing gas, to appoint some afternoon in the week and then take out teeth for as many as had made appointments. I find from minutes, that on July 26th, 1845, seven were extracted while the name of only two of the individuals were recorded."

Dr. John B. Terry, dentist, of Hartford, Conn.: "I had an office adjoining the one usually occupied by the late Dr. Wells; and we were associated together on the 19th of December, 1846, in the practice of dentistry. For nearly a year before this we were associated without terms of partnership, and while he was absent I attended to his business in part, and made him an allowance. My impression is that Dr. Wells used the gas while attending to business, and when he was absent I administered the gas for him. I am certain that prior to October, 1846, I was in the frequent habit of administering the gas, and considered it then as I do now, as more useful than any anæsthetic agent for the purposes of dentistry. * * * I think I have administered more of this gas for dental purposes, than any other person, and I am well acquainted with all its effects."

John Braddock, of Hartford, dentist: "In the spring of 1845, I saw several teeth extracted from different persons under the influence of this agent (meaning the nitrous oxyd) by Dr. John M. Riggs, with the most satisfactory results. The patients seemed to experience no pain whatever, and after the operations were performed and the effects of the gas had passed away, they so expressed themselves."

Dr. E. E. Crowfoot, of Hartford, dentist: "I have had some personal experience in the use of anæsthetic agents, having extracted two teeth for Miss Angelina Griswold, of West Hartford, while under the influence of nitrous oxyd gas. Both teeth were removed at one sitting and in a satisfactory manner."

This, the witness says, was previous to a severe sickness which he had, commencing in September, 1846.

P. W. Ellsworth, M. D., son of Ex-Governor Ellsworth, and grandson of the distinguished Oliver Ellsworth: "Very shortly before or after the visit of Dr. Wells to Boston, with a view to bring out his discovery, (to wit: in January, 1845,) I witnessed a successful dental operation, being the extraction of a tooth without pain, by administering nitrous oxyd gas."

The witness adds, that he thinks that both Dr. Wells and Dr. Riggs were present, but that he can not tell which performed the operation.

"Some time in the year 1845 or 1846, according to my best recollection, and before Dr. Morton's pretensions to this discovery were advanced, though I will not be positive as I may be mistaken, I extracted a tooth for Mrs. Webb, then of Middletown, in this State, but now the wife of Professor Benjamin Silliman, of New Haven, administering the nitrous oxyd gas. The operation was unattended with pain and was entirely successful."

E. E. Marcy, M. D., of the city of New York: This witness after stating in substance a conversation between Dr. Wells and himself, during which he (Dr. W.) spoke of the availability of the nitrous oxyd as an anæsthetic agent, proceeds as follows: "I therefore expressed some doubt to

Dr. Wells when he announced the above fact. In reply, he said 'I am about to extract a tooth under its influence, and if you will go to my office, I will demonstrate to you the truth of my statement.' Accordingly, on the same day I went to his office and witnessed the extraction of a tooth from F. C. Goodrich, Esq., of said Hartford, by Dr. Wells, after nitrous oxyd gas had been inhaled and without the slightest consciousness of pain on the part of the gentleman operated on. Not only was the extraction accomplished without pain, but the inhalation of the gas was effected without any of those indications of excitement or attempts at muscular exertion, which is so commonly obtained when the gas is administered without a definite object or previous mental preparation. * * * * It was in the fall of 1844, I am positive, and within two or three days after I had understood Dr. Wells had made the discovery."

5. These witnesses were not any of them the subjects of the anæsthetic operation, but either operated themselves or were present at and speak of the operations of others, but it is quite time to call up the parties themselves, who were brought under the influence of nitrous oxyd.

Francis C. Goodrich, of Hartford: "I submitted to the operation of having a tooth extracted by Dr. Wells, while under the influence of nitrous oxyd gas, which was performed in the presence of Drs. Marcy, Kitteridge, and Riggs, and was unattended with even the slightest sensation of pain." This he says was in the latter part of the year 1844.

John Gaylord Wells, of Hartford: "Dr. Wells extracted a tooth for me immediately after the extraction of his own. It was certainly in the month of December, 1844. The gas was given from a large bag, on this occasion. I had one tooth removed and afterward a number at different times, and all without pain."

William H. Burleigh, of Hartford, says, that witnessing these operations, "I was so delighted and surprised with its manifest success, that I desired a trial of it upon myself. The gas was accordingly administered, and two carious teeth were extracted from my lower jaw without the least suffering on my part, though ordinarily owing to the firmness with which my teeth are fixed in my jaw, I suffer extreme pain from their extraction."

But "by the mouth of two or three witnesses, every word shall be established." Why then make any further extracts from numerous other depositions brought forward by Mr. Smith in this connection. If these are not adequate to overcome incredulity, it will be useless to quote more.

6. But I shall not do justice to the claim of Dr. Wells without laying before your readers proofs of his efforts to make anæsthesia practical in dentistry other than those already described. I have thus far spoken of actual operations, but Dr. Wells in the meantime was doing much more,—he was incessantly exerting himself to improve the agent and the instrument or means to be used in exhibiting it to his patients.

P. W. Ellsworth, M. D.: "I know that Dr. Wells from the time of his discovery up to the time of his death, was making improvements both in the preparation and mode of administering the gas, and ultimately it became in his hands more efficient than it was in the first instance. The gas was much more pure, and the instruments were better."

John B. Terry, dentist: "During the time he was engaged in his profession, he continued to make improvements in the construction of his inhaling apparatus, in the nitrate of ammonia, by which the gas was made, in the gas itself, and its mode of preparation from the time of his discovery to his death."

Ah! what of that abandonment of not only the nitrous oxyd, but of anæsthesia in toto! of which Col. Bissell speaks in his report?

7. But I wish to bring to the notice of your readers proofs touching some of the characteristics of this remarkable man.

John M. Riggs, dentist: "Dr. Wells was enthusiastic and sanguine in the pursuit of objects toward which he turned his attention."

E. E. Marcy, M. D.: "Dr. Wells was exceedingly enthusiastic upon the subject—was incessantly conversing about it, and prosecuting his experiments. * * * * He was a man of strict rectitude, and in every way worthy of entire confidence. He possessed a peculiarly active, investigating and philosophical mind, and was therefore almost constantly engaged in researches and enquiries such as would naturally attract the attention of a man of his taste."

P. W. Ellsworth, M. D.: "I further say that I had many conversations with Dr. Wells, on the subject of his discovery in 1845 and 1846, and indeed up to the time of his death, and he was at all times enthusiastic in regard to it, and I did not know or suspect that any one controverted the right of Dr. Wells, until Dr. Morton advanced his claim in 1846."

John B. Terry, dentist: "Dr. Wells' confidence in the gas was constantly increasing from the first; no one, to my knowledge, doubted that Wells was the discoverer of the anæsthetic properties of the gas. nor did I hear at that time, that any one claimed to be the discoverer but him."

8. It must be obvious that such a discovery could not have been made, and carried into practice so extensively without the facts having become perfectly notorious at Hartford.

John M. Riggs, dentist: "It was a subject of profound interest in Hartford, and attracted universal attention through the years 1845 and 1846."

E. E. Marcy, M. D.: "Immediately after the discovery, the fact became generally known in Hartford, and was the subject of much conversation."

P. W. Ellsworth, M. D.: "Very soon after Dr. Wells made the above discovery, the fact became generally known in this community and was the subject of much conversation, and Dr. Wells was universally reported to be its originator or author, and has ever since been and is now believed here to be entitled to the credit thereof."

John Braddock, of Hartford: "The discovery of Dr. Wells was notorious in Hartford at that time," (1845,) "it was a common topic of conversation, and I have no hesitation in saying, that in my opinion Dr. Wells was the first to discover and use an agent by means of which dental and surgical operations could be performed without pain."

Hon. James Dixon, United States Senator from Connecticut: "I would add, that the discovery of Dr. Wells was notorious in Hartford, in the spring of 1845, and was then and for some time had been and continued to be a frequent topic of conversation. It excited great attention, and was deemed of much importance."

Here, Mr. Editor, I must rest for the present. I trust that your readers will be prepared to admit that I have made some progress toward a realization of my promise that I would show conclusively where we are to look for the true author of modern anæsthesia. But I should do injustice to the cause I vindicate, were I to ask them to render a verdict and enter up judgment here, for I have several other most interesting chapters to open and lay before them, with contents not less significant than those heretofore and now communicated. Perhaps I may require an interval for other avocations, of one or two numbers of your valuable journal before I resume my pen. In the meantime permit me to bid you and your readers a respectful adieu.—*Medical & Surg. Reporter.*

